# Impact of Smoking and Other Lifestyle Factors on Life Expectancy among Japanese: Findings from the Japan Collaborative Cohort (JACC) Study

**DOI:** 10.2188/jea.JE20100017

**Published:** 2010-09-05

**Authors:** Akiko Tamakoshi, Miyuki Kawado, Kotaro Ozasa, Koji Tamakoshi, Yingsong Lin, Kiyoko Yagyu, Shogo Kikuchi, Shuji Hashimoto

**Affiliations:** 1 Department of Public Health, Aichi Medical University School of Medicine, Aichi, Japan; 2 Department of Hygiene, Fujita Health University School of Medicine, Aichi, Japan; 3 Department of Epidemiology, Radiation Effects Research Foundation, Hiroshima, Japan; 4 Department of Nursing, Nagoya University School of Health Sciences, Nagoya, Japan

**Keywords:** lifestyle, smoking, cohort study, life expectancy

## Abstract

**Background:**

A number of lifestyle factors, including smoking and drinking, are known to be independently associated with all-cause mortality. However, it might be more effective in motivating the public to adopt a healthier lifestyle if the combined effect of several lifestyle factors on all-cause mortality could be demonstrated in a straightforward manner.

**Methods:**

We examined the combined effects of 6 healthy lifestyle behaviors on all-cause mortality by estimating life expectancies at 40 and 60 years of age among 62 106 participants in a prospective cohort study with a 14.5-year follow-up. The healthy behaviors selected were current nonsmoking, not heavily drinking, walking 1 hour or more per day, sleeping 6.5 to 7.4 hours per day, eating green leafy vegetables almost daily, and having a BMI between 18.5 to 24.9.

**Results:**

At age 40, we found a 10.3-year increase in life expectancy for men and a 8.3-year increase for women who had all 6 healthy behaviors, as compared with those who had only 0 to 2 healthy behaviors. Increases of 9.6 and 8.2 years were observed for men and women, respectively, at age 60 with all 6 healthy behaviors. When comparing currently nonsmoking individuals with 0 to 1 healthy behaviors, the life expectancy of smokers was shorter in both men and women, even if they maintained all 5 other healthy behaviors.

**Conclusions:**

Among individuals aged 40 and 60 years, maintaining all 6 healthy lifestyle factors was associated with longer life expectancy. Smokers should be encouraged to quit smoking first and then to maintain or adopt the other 5 lifestyle factors.

## INTRODUCTION

Unhealthy lifestyle behaviors such as cigarette smoking,^1^^,^^2^ excessive alcohol drinking,^3^^,^^4^ physical inactivity,^5^ too much or too little sleep,^6^^,^^7^ low consumption of green leafy vegetables,^8^^,^^9^ and overweight^10^^,^^11^ are associated with increased risk of all-cause mortality. However, because these lifestyle factors are mutually related,^12^^,^^13^ it is important to investigate their combined effects in a straightforward way. We recently reported the combined effects of modifiable lifestyle factors on all-cause mortality.^14^ As compared with people with 0 to 2 healthy lifestyle behaviors (current nonsmoking, not heavily drinking, walking 1 hour or more per day, sleeping 6.5 to 7.4 hours per day, eating green leafy vegetables almost daily, and a body mass index (BMI) between 18.5 to 24.9), men and women with 6 healthy behaviors had 58% and 51% respective reductions in the risk of all-cause mortality.

Among these behaviors, smoking may be the best known risk factor. Studies have suggested that smoking has a larger impact on all-cause mortality than other factors. The EPIC-Norfolk Prospective Population Study found that the relative risks of all-cause mortality were 1.77 for smoking, 1.24 for physical activity, 1.26 for alcohol intake, and 1.44 for vitamin C level among men and women aged 45 to 79 years.^15^ Similarly, ever-smoking women had a 1.66 relative risk in the Nurse’s Health Study, whereas the risks associated with other factors (high BMI, physical activity, diet, and alcohol intake) were less than 1.35.^16^ Thus, it is useful to investigate the discrete effects of smoking status and other lifestyle factors on all-cause mortality.

In order to motivate the public to make lifestyle modifications, it is necessary to show the combined impact of lifestyle factors on health in a manner that is easy to understand. Life expectancy indicates the theoretical number of years of life remaining at a given age, and is a well-known, comprehensive value for representing the health status of a population. The concept of life expectancy is understandable to the public and can be readily used by health providers to promote evidence-based health plans. Thus, our objective was to estimate life expectancies according to the number of healthy lifestyle behaviors at 40 and 60 years of age among participants in the Japan Collaborative Cohort (JACC) Study. We also separately estimated life expectancies for current smokers and current nonsmokers.

## METHODS

### Study subjects and data collection

The study design and methods of the JACC Study have been described previously.^17^^,^^18^ Briefly, the cohort was assembled from 1988 to 1990 by using a self-administered questionnaire; it ultimately enrolled 110 792 subjects (46 465 men and 64 327 women) aged 40 to 79 years. The cause and date of death among the study subjects were identified by reviewing all death certificates in each area with the permission of the Director-General of the Prime Minister’s Office (Ministry of Internal Affairs and Communications). Those who moved out of a study area were regarded as censored. We followed the subjects until the end of 2006, except in the 7 areas where follow-up was discontinued at the end of 1999 or 2003. The Ethical Board of Nagoya University School of Medicine, where the central office of the JACC Study is located, approved the entire study design.

### Lifestyle variables

We selected 6 lifestyle behaviors: smoking status, alcohol consumption, walking duration, sleep duration, consumption of green leafy vegetables, and BMI. The selection of these lifestyle factors was based on the results of our previous cohort study and on an extensive review of other epidemiologic studies that had investigated the relationship between lifestyle factors and mortality; details of the selection and dichotomization of these lifestyle factors are described elsewhere.^14^ Briefly, the potential health status of each factor was: current nonsmoking (including former smokers),^1^^,^^19^ drinking no more than 1 *gou* (23 g of alcohol) per occasion or current nondrinking (including former drinkers),^4^ walking 1 hour or more per day,^20^^,^^21^ sleeping 6.5 to 7.4 hours per day,^6^^,^^7^ eating green leafy vegetables almost daily,^8^^,^^22^ and desirable weight for height (BMI between 18.5 to 24.9).^11^^,^^23^ The number of healthy lifestyle behaviors for each subject was summed, with a higher number indicating a more healthful lifestyle.

The 62 106 subjects (27 582 men and 34 524 women) with complete information on all 6 lifestyle behaviors were considered eligible for the analyses.

### Analysis

Age- and sex-specific mortality rates within 5-year age categories were calculated based on the person-year method according to the number of healthy lifestyle behaviors. Calculation of mortality was started at age 40 years and the last category was age 85 years or older. Because there was a small number of subjects included in the category at age 90 or older, mortality at age 85 years or older was calculated with the assumption that the ratio of mortality at age 85 to 89 years to mortality at age 85 years or older was the same in this cohort as in the general Japanese population. Thus, mortality at age 85 years or older in the study was calculated as MR_85+_ = MR_85–89_ × JMR_85+_/JMR_85–89_, where MR*_x_* indicates mortality at age *x* in the JACC Study, and JMR*_x_* indicates mortality at age *x* throughout Japan in 2000. Chiang’s abridged life table method^24^ was used to calculate life expectancies with respect to the number of healthy behaviors. The fraction of all age categories of life was used to construct an abridged life table.^24^^,^^25^ Those fractions were calculated from a complete life table for the year 2000 in Japan. Confidence intervals for life expectancies were also calculated using the formulae proposed by Chiang. Additionally, we estimated life expectancies according to the number of healthy behaviors stratified by smoking status (current smoking or current nonsmoking at baseline). The age-adjusted mortality rates according to the number of healthy lifestyle behaviors were calculated based on the 5-year age-specific mortality by sex, with the whole cohort considered as the standard population. We used SAS Ver. 9.1.3 (SAS Institute Inc., Cary, NC, USA) for analyses at the Aichi Medical University Computation Center.

## RESULTS

During an average follow-up period of 14.5 years, a total of 10 843 deaths occurred by 2006 (6633 men and 4210 women). Death from any cancer accounted for 38.5% of deaths in men and 34.0% of deaths in women, and death from cardiovascular disease accounted for 27.6% and 32.8% of deaths in men and women, respectively.

At baseline, there were 16 363 current smokers: 14 663 men (53.2% of men) and 1700 women (4.9% of women). Current nonsmokers were older than current smokers among both men and women. Also, the former were more educated, had less mental stress, were more likely to have a spouse, and were more likely to eat breakfast every day. Among women, the proportion of those with a history of stroke, myocardial infarction, or cancer was lower among current nonsmokers; however, the proportion was higher among men. With regard to the number of healthy lifestyle behaviors other than nonsmoking, both men and women with a higher number of healthy behaviors were more likely to eat breakfast every day than men and women with a lower number of such behaviors. The former were also more educated (except for smoking men), older and had less mental stress (in both smoking and nonsmoking men), and were more likely to have a spouse (in currently nonsmoking women).

Age-adjusted mortality rates by number of healthy lifestyle behaviors are shown in Table 1 (the mortality rate of smoking women with 5 healthy lifestyle behaviors could not be calculated because the sample size was too small). The rate varied from 8.9 to 20.7 per 1000 men and from 6.2 to 11.6 per 1000 women. Age-adjusted mortality rates in currently smoking men and women were 20.4 and 13.0 per 1000, respectively; among current nonsmokers, the rates were 13.9 and 8.1.

**Table 1.** Age-adjusted mortality rates according to number of healthy lifestyle behaviors

		Men				Women			
			
		Number	Person-years	Deaths	Age-adjusted mortality (per 1000)	Number	Person-years	Deaths	Age-adjusted mortality (per 1000)
No. of healthy lifestyle behaviors									
	0–2	5092	141 967	2478	20.7	2277	39 055	500	11.6
	3	9147	125 658	2133	16.9	9014	131 480	1292	9.4
	4	8969	85 414	1464	14.9	13 147	189 491	1466	7.9
	5	3694	31 931	494	12.6	8287	120 141	795	7.0
	6	680	5587	64	8.9	1799	26 550	157	6.2
	
	Overall	27 582	390 504	6633	17.0	34 524	506 720	4210	8.3

No. of healthy lifestyle behaviors other than smoking									
​ Current smoker									
	0–1	2908	40 953	706	22.9	202	2668	44	19.1
	2	4980	70 007	1309	22.2	527	7171	103	14.5
	3	4711	67 370	1179	18.6	600	8708	91	11.7
	4	1763	25 546	443	18.1	322	4669	39	9.1
	5	301	4315	80	18.7	49	—	9	—
	
	Overall	14 663	208 184	3717	20.4	1700	23 940	286	13.0
	
​ Current nonsmoker									
	0–1	2184	31 012	463	15.4	2075	29 213	353	10.5
	2	4167	58 289	954	15.0	8487	122 771	1201	9.3
	3	4258	59 873	1021	13.9	12 547	184 822	1427	7.8
	4	1931	27 617	414	11.6	7965	119 413	786	7.0
	5	379	5587	64	8.9	1750	26 550	157	6.2
	
	Overall	12 919	182 358	2916	13.9	32 824	482 760	3924	8.1

Table 2 shows life expectancies at 40 and 60 years of age. Life expectancies among 40-year-old men and women were 39.9 and 46.5 years, respectively, in those with 0 to 2 healthy behaviors, and 50.2 and 54.8 years in those with all 6 healthy lifestyle behaviors. Life expectancy rose as the number of healthy behaviors increased. The same life expectancy trend was observed in 60-year-old men and women. The differences in life expectancy between those with 6 healthy behaviors and those with only 0 to 2 such behaviors were 10.3 and 8.3 years in men and women, respectively, at 40 years of age, and 9.6 and 8.2 years at 60 years of age.

**Table 2.** Estimated life expectancies according to number of healthy lifestyle behaviors

		Men				Women			
			
		Life expectancy at 40 years of age		Life expectancy at 60 years of age		Life expectancy at 40 years of age		Life expectancy at 60 years of age	
			(95% CI)		(95% CI)		(95% CI)		(95% CI)
No. of healthy lifestyle behaviors									
	0–2	39.9	(39.5–40.3)	21.9	(21.6–22.2)	46.5	(45.5–47.4)	28.1	(27.5–28.7)
	3	42.3	(41.9–42.8)	23.9	(23.6–24.2)	48.0	(47.6–48.4)	29.1	(28.8–29.4)
	4	43.4	(42.8–44.1)	25.1	(24.7–25.5)	50.1	(49.8–50.4)	30.9	(30.6–31.2)
	5	44.7	(43.8–45.7)	25.9	(25.4–26.5)	50.9	(50.5–51.2)	31.5	(31.2–31.8)
	6	50.2	(48.1–52.3)	31.5	(29.8–33.2)	54.8	(53.3–56.2)	36.3	(35.4–37.1)
	
	Overall	42.1	(41.8–42.3)	23.8	(23.6–24.0)	49.5	(49.2–49.7)	30.4	(30.2–30.6)

No. of healthy lifestyle behaviors other than smoking									
​ Current smoker									
	0–1	39.0	(38.3–39.7)	21.0	(20.4–21.5)	39.7	(36.8–42.7)	22.9	(20.8–25.0)
	2	39.0	(38.5–39.6)	21.3	(20.9–21.7)	44.0	(42.0–45.9)	25.4	(24.1–26.7)
	3	41.2	(40.6–41.9)	23.0	(22.6–23.4)	46.6	(45.0–48.1)	27.7	(26.3–29.1)
	4	42.0	(41.0–42.9)	23.2	(22.5–23.9)	48.1	(45.9–50.3)	29.3	(27.5–31.0)
	5	41.4	(39.3–43.4)	23.2	(21.4–24.9)	—		—	
	
	Overall	40.1	(39.8–40.5)	22.1	(21.8–22.3)	44.9	(43.9–45.9)	26.4	(25.7–27.2)
	
​ Current nonsmoker									
	0–1	43.4	(42.7–44.2)	24.5	(23.9–25.1)	47.9	(46.8–49.0)	29.3	(28.6–30.0)
	2	43.6	(43.0–44.2)	24.9	(24.5–25.3)	48.1	(47.6–48.5)	29.2	(28.9–29.5)
	3	43.9	(43.1–44.7)	25.8	(25.3–26.2)	50.1	(49.8–50.5)	30.9	(30.7–31.2)
	4	45.3	(44.2–46.4)	26.4	(25.8–27.0)	50.9	(50.5–51.2)	31.5	(31.2–31.8)
	5	50.2	(48.1–52.3)	31.5	(29.8–33.2)	54.8	(53.3–56.2)	36.3	(35.4–37.1)
	
	Overall	44.2	(43.8–44.5)	25.6	(25.3–25.8)	49.7	(49.5–49.9)	30.6	(30.4–30.8)
	
All Japan (2000)^35^									
		39.1		21.4		45.5		26.9	

The trend toward longer life expectancy with a larger number of healthy behaviors was observed even when subjects were stratified according to smoking status. Among smoking women, the life expectancy of those with 5 healthy behaviors could not be calculated because the sample size was too small and no deaths were observed in their category at age 85 to 89 years. As compared with their currently nonsmoking counterparts, both male and female current smokers had shorter life expectancies at baseline. On average, the differences were 4.0 and 4.8 years at 40 years of age in men and women, respectively, and 3.5 and 4.2 years at 60 years of age. Among male smokers at ages 40 and 60, even when all 5 other healthy behaviors were present, their life expectancies were still shorter than those of nonsmokers with 0 to 1 other healthy behaviors. Similarly, smoking women with 4 of the other healthy behaviors had a shorter life expectancy at age 60, as compared with nonsmoking women with 0 to 1 healthy behaviors other than nonsmoking, although the life expectancy of the former at age 40 was only slightly longer (0.1 years) than that of the latter. Moreover, the differences between subjects with 5 healthy behaviors and those with 0 to 1 healthy behaviors were smaller among smokers than among nonsmokers, except for women at 40 years of age. The differences in life expectancy according to the number of healthy lifestyle behaviors in smoking men were 2.4 and 2.2 years at 40 and 60 years of age, respectively; the respective differences were 6.8 and 7.0 years in nonsmoking men.

## DISCUSSION

The current study presents life expectancies according to the number of healthy lifestyle behaviors, using data from a large-scale population-based cohort followed for 14.5 years. The results showed that life expectancy rises as the number of healthy behaviors increases. The life expectancy of subjects maintaining 6 healthy lifestyle behaviors was 10.3 and 8.3 years longer than those with 0 to 2 healthy lifestyles in men and women, respectively, at age 40 years, and 9.6 and 8.2 years at age 60. Smoking status had the greatest impact on life expectancy. The life expectancies of male and female smokers who maintained all other healthy lifestyle factors were nevertheless shorter than those of their currently nonsmoking counterparts with only 0 to 1 healthy lifestyle behaviors. Furthermore, the increases in life expectancy associated with having more healthy behaviors were smaller among smokers than among nonsmokers, particularly in men.

Studies have investigated the combined effects of lifestyle factors on all-cause mortality by calculating life expectancies. Stamler et al reported that life expectancy for low-risk subcohorts (ie, those with favorable levels of cholesterol and blood pressure, nonsmokers, nondiabetics, and those with no MI or ECG abnormalities) was 5.8 to 9.5 years higher than that of others in 5 large US cohorts.^26^ Based on an 11-year follow-up of UK cohorts aged 45 to 79 years, Khaw et al estimated that the mortality risk for individuals with 4 healthy behaviors (current nonsmoker, not physically inactive, moderate alcohol intake, and plasma vitamin C >50 mmol/l), as compared with individuals with none of these behaviors, was equivalent to being 14 years younger in chronological age.^15^ Although the investigated lifestyle factors varied in these reports, they all observed differences in life expectancy between subjects with healthy lifestyle behaviors and those without them.

In Japan, the difference in life expectancy due to smoking status was previously calculated based on NIPPON DATA80 by Murakami et al.^27^ Life expectancy in men and women aged 40 years was 42.1 and 45.6 years, respectively, in never smokers, 40.4 and 45.9 years in ex-smokers, and 38.6 and 43.4 years in current smokers, which represent slightly smaller differences in life expectancies between smokers and nonsmokers than those noted in our study. However, studies from other developed countries found larger differences in the life expectancies of smokers and nonsmokers. The life expectancies of 50-year-old never smokers and current smokers were 32.6 and 26.3 years in men in the Whitehall Study, England.^28^ In the Framingham Study, the differences in life expectancy between never smokers and always smokers were 8.7 years in men and 7.6 years in women at age 50.^29^ While all these studies found longer life expectancies among nonsmokers than current smokers, the differences varied. The different circumstances of smokers may explain this variation. In Japan, at the time of our baseline survey, smoking was very common in Japanese men, and thus, some never-smoking men might have had health problems that compelled them to avoid smoking. Such unhealthy conditions among nonsmoking men might reduce the difference in life expectancies between smokers and nonsmokers. In contrast, smoking by women was not common at that time, especially among housewives. Thus, some smoking women might have been healthier than others.^27^ This could have led to prolonged life span among smoking women, and might have diminished the difference in life expectancies between smokers and nonsmokers. These may partially explain why the differences in life expectancies in Japan were smaller than those reported in other developed countries. However, the prevalence of current smokers in our study was 53.2% in men, which was lower than the prevalence noted in NIPPON DATA80 (62.9%), which indicates a possible reduction in the number of unhealthy subjects among nonsmokers. This could account for the relatively larger differences in life expectancies among men in our study, as compared with NIPPON DATA80. The reason for the larger differences in life expectancies among women was not clear, as the prevalence of current smokers was lower in our study than in NIPPON DATA80 (4.9% and 8.8%, respectively). However, the distribution of other behaviors may be different.

This study revealed that the life expectancies of male and female smokers were shorter than those of nonsmokers with 0 to 1 healthy lifestyle behaviors, even when the former maintained all other healthy behaviors. In addition, the increase in life expectancy attributable to having a higher number of healthy lifestyle behaviors was smaller among current smokers than among nonsmokers, particularly in men. Two previous studies estimated the effect of smoking on life expectancies separately from other lifestyle factors. As compared with male never smokers reporting low physical activity, the expected age at death at age 65 for highly active male ever smokers was slightly older, but that for moderately active male ever smokers was younger^30^; the result was the same for women. Regarding obesity, Peeters et al found that the life expectancy of 40-year-old male smokers of normal weight was 1 year shorter than that of obese nonsmokers; although a similar such reduction in life expectancy was not observed among women.^31^ These studies suggest that smoking has a somewhat larger impact on life expectancy than physical activity or obesity. Our results offer more evidence regarding the impact of smoking on health.

The lifestyle factors chosen in this study were current nonsmoking, not heavily drinking, walking 1 hour or more per day, sleeping 6.5 to 7.4 hours per day, eating green leafy vegetables almost daily, and BMI between 18.5 to 24.9. We previously found a clear reduction in mortality risk with an increasing number of these 6 healthy lifestyle behaviors.^14^ These factors were selected based on the results from our previous cohort study^4^^,^^14^^,^^22^^,^^32^^–^^34^ and a review of epidemiologic studies^1^^,^^3^^,^^6^^,^^8^^,^^11^^,^^20^ that included data on associations between lifestyle factors and mortality. The lifestyle factors selected in this study are key elements of a healthy lifestyle included in *Health Japan 21*, a recent health promotion initiative of the government of Japan. Selected factors were dichotomized into healthy or unhealthy, and the healthy behaviors of each subject were summed. Therefore, the factors we selected for the study were sufficiently reliable, easily understood, and easy to calculate.

Our results should serve as a useful tool to help both the general public and health planners to understand the importance of maintaining a healthy lifestyle. Life expectancy is a readily comprehensible value that allows us to use differences in survival age to represent the combined risk impacts of different behaviors. With these easily understandable data, health planners are better able to encourage lifestyle changes in people. For a person who is considering a lifestyle change for health reasons, knowing how many years of life one might gain could be a powerful incentive.

The strengths of our study were 1) that it included data from a large cohort of subjects from all over Japan (including more than 10 000 deceased), and 2) its long follow-up period of approximately 14.5 years. These advantages allowed us to estimate life expectancies according to a number of healthy lifestyle behaviors. Moreover, we were able to assess the impact of smoking status on life expectancy separately from other lifestyle factors. As a result, it is clear that smoking has a larger impact than other lifestyle factors on life expectancy.

Our results show longer life expectancies that those noted in the Japanese complete life table at 2000^35^: 2.9 and 3.9 years longer in men and women, respectively, at the age of 40, and 2.4 and 3.5 years at the age of 60. People with health problems at baseline were not included in this study, and subjects who moved away from the study area for any reason were excluded from our analysis at that time. Therefore, age-specific mortality rates moved lower, which may explain why estimated life expectancies in this study were longer than those of the general population of Japan. Because such an overestimation might occur randomly, the rank of life expectancies among the lifestyle groups in this study should prove representative of the general population of Japan.

There were some limitations in this study that warrant discussion. First, as lifestyle status was self-reported, some measurement errors may have occurred. However, the JACC Study was a prospective cohort study, and misclassification of health status was more likely to be random. Second, data were only collected at baseline, and subsequent behavior changes could not be taken into account in our analyses. Kawado et al^36^ compared baseline data with interim data collected approximately 5 years later in some of our participants, and found that the proportions of smokers and drinkers had decreased. If such behavioral changes occurred in other lifestyle factors, each change that resulted in a healthier lifestyle might diminish the differences between healthy and unhealthy groups at baseline, and the differences between estimated life expectancies might be diminished due to these changes. Third, we did not consider the effects of factors other than the 6 healthy lifestyle factors investigated in this report. Of course, not all differences in life expectancy were caused by these 6 lifestyle factors. However, because lifestyle factors are mutually related, it is possible that the number of healthy lifestyle behaviors is not merely a given number, and that estimated life expectancies in this study somewhat reflect the effects of other lifestyle factors.

In conclusion, we estimated life expectancy with respect to 6 healthy lifestyle behaviors, and found that men and women with all these behaviors had 10.3 and 8.3 years, respectively, of additional life expectancy at age 40 years, as compared with those with 0 to 2 such behaviors, and 9.6 and 8.2 years of additional life expectancy at age 60. Even though adoption of any of these behaviors would likely improve life expectancy, smoking status had the largest impact. Smoking cessation is thus the first and best way to extend life expectancy.

### Members of the JACC Study Group

The present members of the JACC Study Group, and their affiliations, are: Dr. Akiko Tamakoshi (present chairperson of the study group), Aichi Medical University School of Medicine; Drs. Mitsuru Mori & Fumio Sakauchi, Sapporo Medical University School of Medicine; Dr. Yutaka Motohashi, Akita University School of Medicine; Dr. Ichiro Tsuji, Tohoku University Graduate School of Medicine; Dr. Yosikazu Nakamura, Jichi Medical School; Dr. Hiroyasu Iso, Osaka University School of Medicine; Dr. Haruo Mikami, Chiba Cancer Center; Dr. Michiko Kurosawa, Juntendo University School of Medicine; Dr. Yoshiharu Hoshiyama, University of Human Arts and Sciences; Dr. Naohito Tanabe, Niigata University School of Medicine; Dr. Koji Tamakoshi, Nagoya University Graduate School of Health Science; Dr. Kenji Wakai, Nagoya University Graduate School of Medicine; Dr. Shinkan Tokudome, National Institute of Health and Nutrition; Dr. Koji Suzuki, Fujita Health University School of Health Sciences; Dr. Shuji Hashimoto, Fujita Health University School of Medicine; Dr. Shogo Kikuchi, Aichi Medical University School of Medicine; Dr. Yasuhiko Wada, Faculty of Human Life and Environmental Science, Kochi Women’s University; Dr. Takashi Kawamura, Kyoto University Center for Student Health; Dr. Yoshiyuki Watanabe, Kyoto Prefectural University of Medicine Graduate School of Medical Science; Dr. Kotaro Ozasa, Radiation Effects Research Foundation; Dr. Tsuneharu Miki, Kyoto Prefectural University of Medicine Graduate School of Medical Science; Dr. Chigusa Date, Faculty of Human Environmental Sciences, Nara Women’s University; Dr. Kiyomi Sakata, Iwate Medical University; Dr. Yoichi Kurozawa, Tottori University Faculty of Medicine; Dr. Takesumi Yoshimura, Fukuoka Institute of Health and Environmental Sciences; Dr. Yoshihisa Fujino, University of Occupational and Environmental Health; Dr. Akira Shibata, Kurume University School of Medicine; Dr. Naoyuki Okamoto, Kanagawa Cancer Center; and Dr. Hideo Shio, Moriyama Municipal Hospital.
